# Heart Rate Estimation Considering Reconstructed Signal Features Based on Variational Mode Decomposition via Multiple-Input Multiple-Output Frequency Modulated Continuous Wave Radar

**DOI:** 10.3390/s24216809

**Published:** 2024-10-23

**Authors:** Sara Nakatani, Mondher Bouazizi, Tomoaki Ohtsuki

**Affiliations:** 1 Graduate School of Science and Technology, Keio University, Yokohama 223-8522, Japan; nakatani@ohtsuki.ics.keio.ac.jp; 2 Faculty of Science and Technology, Keio University, Yokohama 223-8522, Japan; bouazizi@ohtsuki.ics.keio.ac.jp

**Keywords:** heart rate estimation, MIMO FMCW radar, VMD

## Abstract

Accurate heart rate estimation using Doppler radar and Frequency Modulated Continuous Wave (FMCW) radar is highly valued for privacy protection and the ability to measure through clothing. Conventional methods struggle to isolate the heartbeat from respiration and body motion. This paper introduces a novel heart rate estimation method using Variational Mode Decomposition (VMD) via Multiple-Input Multiple-Output (MIMO) FMCW radar. The proposed method first estimates human positions within the radar’s coverage, reducing noise by focusing on signals from these positions. The signal is then decomposed into multiple Intrinsic Mode Function (IMF) signals using VMD, and the heartbeat-specific IMF is extracted based on its center frequency. The heart rate signal is reconstructed using weighted addition of IMF signals for each radar cell, with cells defined by specific angles and distances within the coverage area. Peak detection is used to estimate heart rate from these reconstructed signals. To ensure accuracy, the method selects the heart rate estimate with the highest energy and periodicity for the first four time windows. From the fifth time window onward, it selects the estimate closest to the average of the previous four, minimizing extraneous variations. Experiments conducted with one and two subjects showed promising results. In case 1, with one subject, the method achieved a Mean Absolute Error (MAE) of 2.54 BPM and an exclusion rate of 0.94% using MIMO FMCW radar, compared to 4.72% with Doppler radar. In case 2, with two subjects, the method achieved an MAE of 2.28 BPM, confirming accurate simultaneous heart rate estimation.

## 1. Introduction

Vital sign monitoring has attracted attention in the fields of hospital and home health care because of its usefulness in understanding a person’s health status [1,2,3,4]. In particular, heart rate variability is an important biological indicator that reflects the condition of one’s health [5]. Monitoring heart rate variability makes it possible to detect signs of illness, fatigue, and stress [6].

There are contact and non-contact methods for heartbeat detection. While the most common contact-based method, Electrocardiogram (ECG), is the gold standard for clinical heart rate monitoring due to its precision and comprehensive cardiac information, it may be less practical for daily self-checks in non-medical environments. For routine monitoring, non-contact methods offer a more convenient alternative for measuring heart rate and R-R interval (RRI) without the need for attaching electrodes or wearing a device, thus enhancing comfort and ease of use in everyday life. Non-contact heart rate detection methods include methods that use cameras [7,8,9] and radars [10,11,12,13,14,15]. In the studies using cameras [7], the heart rate is estimated from the acquired face images by the camera. Since the heart rate varies with blood pressure, and the hemoglobin in blood has the property of absorbing the green component of light, heart rate is estimated by observing the reflectance of light [8]. Therefore, by performing Independent Component Analysis (ICA) on the color channels of the face image, it is possible to isolate only the color change due to the heartbeat. The disadvantages of such camera-based methods are privacy issues and degradation of detection accuracy in dark environments [9]. In contrast, the use of radars can solve these problems.

To observe heart rate, Doppler radars [10,11,12] and Frequency Modulated Continuous Wave (FMCW) radars [13,14,15] are used. A Doppler radar can observe the velocity and direction of a moving object by transmitting microwaves toward the object and analyzing the reflected Doppler-shifted microwaves [10]. This means that by analyzing the Doppler-shifted received signal reflected from the chest wall, it is possible to capture the chest movement caused by a heartbeat. However, it is difficult to separate signals from different objects when using Doppler radar because the distance information is not available. Furthermore, it is susceptible to noise and body motion in detecting vital signals.

In contrast, FMCW radar transmits microwaves whose frequency is swept linearly toward the target [13]. Based on the difference in frequency between the transmitted and received waves, the distance to the target can be measured. This enables spatial separation of the radar coverage area. Therefore, it is possible to obtain the phase change of the microwaves at the distance where a human is present. In addition to being non-contact, the radar-based heartbeat detection method has the advantages of high privacy and the ability to detect heartbeats while wearing clothes. Because of these advantages, various heart rate detection methods have been developed using Doppler radar [10,11,12] and FMCW radar [13,14,15]. In [11], the spectrogram is calculated based on the received signal and components corresponding to heartbeats are extracted. From the extracted integrated spectrum, given a peak to be judged, peaks before and after are investigated and used to identify whether this peak is attributed to heartbeats. Doing so allows for reducing false detections of peaks attributable to components other than heartbeats. However, it is difficult to extract the heartbeat frequency component if the respiration and heartbeat frequencies are close, or if the respiration and noise components are larger than the heartbeat component. In heartbeat estimation based on the Viterbi algorithm [12], multiple peaks that occur within 0.1 s of the spectral integral are considered to be a single peak candidate, and the average time at which each peak occurs is calculated as the peak candidate occurrence time. From these peak candidates, the Viterbi algorithm is used to detect the peak set that is most likely to be due to a heartbeat. By using the Viterbi algorithm for peak detection in this way, peaks due to heartbeats can be detected with high reliability based on the probability that the peak corresponds to a heartbeat, which is calculated based on the waveform features of the spectral integral value. However, if the time window used for heartbeat estimation is short, the algorithm may become unreliable due to insufficient statistical features. In contrast, in Variational Mode Decomposition (VMD)-based heart rate estimation [14], the chest wall signal is decomposed into multiple frequency modes using VMD and the Intrinsic Mode Function (IMF) signal belonging to each mode is extracted. From the extracted IMF signals, the IMF signal whose center frequency is within the frequency range of the second harmonic signal of the heartbeat is selected. The frequency peaks are then detected from the energy-based weighted reconstructed signal to estimate the heart rate. VMD divides the signal into optimized frequency modes by a combination of center frequency and bandwidth based on the variational principle [15]. This allows for the analysis and extraction of signals in specific frequency bands, thus reducing the influence of factors other than heart rate in the received signal. On the other hand, since the Single-Input Single-Output (SISO) FMCW radar is used, the estimation accuracy may be degraded by the effect of components reflected from objects other than humans. In addition, the second harmonic signal has a smaller amplitude than the fundamental wave, making it difficult to extract the heartbeat component. Furthermore, because the estimated heart rates before and after are not considered, the variation in estimated heart rates in adjacent time windows may be too large.

This paper proposes a heart rate estimation considering reconstructed signal features based on VMD via Multiple-Input Multiple-Output (MIMO) FMCW Radar. By using MIMO FMCW radar, it is possible to estimate the two-dimensional position of a human including distance and angle information, thus reducing the influence of factors attributed to sources other than the humans. MIMO FMCW radar uses beamforming technology to estimate the Direction of Arrival (DoA) and acquire microwaves from the estimated DoA [16]. By analyzing the signal reflected from the estimated DoA, the location of the subject can then be estimated. This means that the heart rate can be calculated based on the phase change at the location of the subject.

The proposed method detects the human location with high accuracy based on the conventional method [17]. The I/Q signals, which represent the in-phase (I) and quadrature (Q) components of a reflected signal, vary in distinctive ways when caused by human motion versus stationary clutter. Human motion typically leads to less correlated variations in I/Q signals compared to stationary objects and background noise. In contrast, the I/Q signal variations from clutter are often highly correlated with signals from nearby locations due to the static nature of the environment. Furthermore, the correlation value between a cell attributed to humans and its two neighboring cells is larger than the correlation value between a cell attributed to local noise and its two neighboring cells. A correlation map showing the correlation between each cell on the range angle map with the power being the CL length of the received I/Q signal is calculated. The human location is selected based on the correlation values and their respective standard deviation on this correlation map. By extracting the signals at these detected locations, only the signals attributed to the person can be used for heart rate detection, which reduces the influence of noise. In addition, using multiple signals corresponding to a human position for heart rate estimation is expected to improve accuracy due to the spatial diversity effect. After acquiring the signals corresponding to the human location, VMD is used to decompose the signals into multiple IMF signals, and only the IMF signals caused by the heartbeat are extracted based on the center frequency of each signal. After extracting the IMF signals caused by heartbeats, reconstructed signals are generated by weighting multiple IMF signals based on their energies, and each signal is used to estimate the heart rate based on peak detection. Finally, the appropriate estimate is selected from the estimated heart rates in multiple cells, taking into account the time transition. This is expected to reduce the extraneous variation in the estimated values.

For comparison, we conducted experiments to observe heartbeats with both the Doppler radar and the MIMO FMCW radar. In the experiments, the subjects were seated and stationary. As for the experimental setting, we assumed two cases: (i) only one subject in the radar coverage area, and (ii) two subjects in the radar coverage area. Using this experimental data, we evaluated the accuracy of the proposed method in estimating heart rate. In addition, we compared the performance of the proposed method with existing heartbeat detection methods.

Through experiments, we confirmed that the proposed method can estimate the heart rate with high accuracy. Specifically, the proposed method was able to estimate heart rate with an average Mean Absolute Error (MAE) of 2.54 Beats Per Minute (BPM) and an average exclusion rate of 0.94 % in case (i), and an average MAE of 2.28 BPM in case (ii).

The rest of this paper is organized as follows. In Section 2, we describe the principle of Doppler radar and MIMO FMCW radar. We then discuss some related work in Section 3. In Section 4, we explain our proposed methods. We evaluate the performance of our methods experimentally in Section 5. Finally, we conclude this paper in Section 6.

## 2. Principle of MIMO FMCW Radar

**Figure 1:**
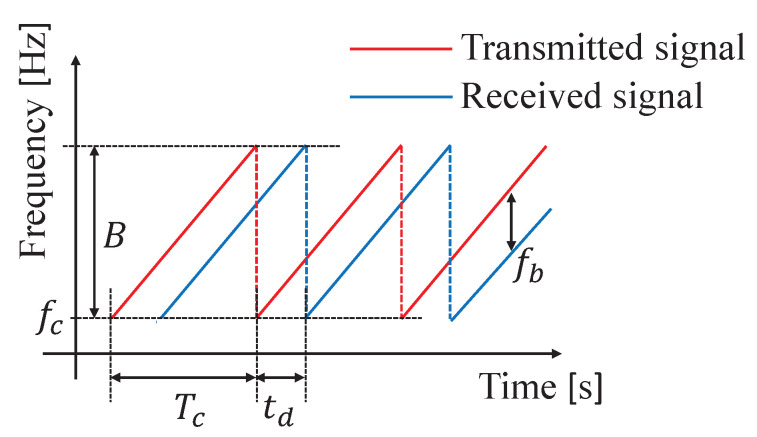
The transmitted and received signals of the used MIMO FMCW radar.

Figure 1 shows the transmitted and received signals of the used MIMO FMCW radar. The FMCW radar transmits a signal called a chirp signal, which is a periodically swept microwave frequency, as shown in Figure 1. Here, $f_{c}$ and *B* represent the minimum sweep frequency and the bandwidth of the chirp, respectively, and $T_{c}$ represents the sweep time. In the FMCW radar system, the transmitted wave is represented by Equation (1).

(1) $$\begin{array}{c} x\left(t\right)=A\cos(2\pi f_{c}t+\pi \frac{B}{T_{c}}t^{2}+\phi \left(t\right)), \end{array}$$

where ϕ(t) is the phase noise. The transmitted microwaves are reflected from the subject. The reflected received signal r(t) is expressed as Equation (2).

(2) $$\begin{array}{c} r\left(t\right)=A\{\cos\left(2\pi f_{c}\left(t-t_{d}\right)+\pi \frac{B}{T_{c}}{\left(t-t_{d}\right)}^{2}+\phi \left(t-t_{d}\right)\right)\}, \end{array}$$

where *A* is the power of the received signal, and $t_{d}$ is given in the following:

(3) $$\begin{array}{c} t_{d}=\frac{2R(t)}{c}, \end{array}$$

where R(t) is the distance between the FMCW radar and the subject including chest motion due to heartbeat, respiration, etc., and *c* is the velocity of the electromagnetic wave. A quadrature mixer is then applied to the received signal, which generates an in-phase signal and a quadrature signal, with a phase difference of π/2 between these signals. The two signals can be expressed as a complex signal y(t) as follows:

(4) $$\begin{array}{c} y\left(t\right)=Ae^{j(2\pi f_{b}t+\Phi \left(t\right)+\Delta \phi \left(t\right))}, \end{array}$$

where $f_{b},\Phi \left(t\right)$, and Δϕ(t) are given as follows:

(5) $$\begin{array}{c} f_{b}=\frac{2BR(t)}{cT_{c}}, \end{array}$$

(6) $$\begin{array}{c} \Phi \left(t\right)=2\pi f_{c}t_{d}+\pi \frac{B}{T_{c}}{t^{2}}_{d}, \end{array}$$

(7) $$\begin{array}{c} \Delta \phi \left(t\right)=\phi \left(t\right)-\phi (t-\frac{2R(t)}{c}). \end{array}$$

Let $R_{\mathrm{const}}$ be the distance between the FMCW radar and the subject, R(t) can be represented approximately as in Equation (8), based on $R_{\mathrm{const}}$ and the relative displacement of the subject, $R_{l}$. Relative displacement $R_{l}$ refers to the movement of the chest due to heartbeat, respiration, etc.

(8) $$\begin{array}{c} R\left(t\right)\approx R_{\mathrm{const}}+R_{l}. \end{array}$$

Therefore, by estimating $R_{\mathrm{const}}$, the phase change due to the relative displacement of the subject at range $R_{\mathrm{const}}$ can be determined.

**Figure 2:**
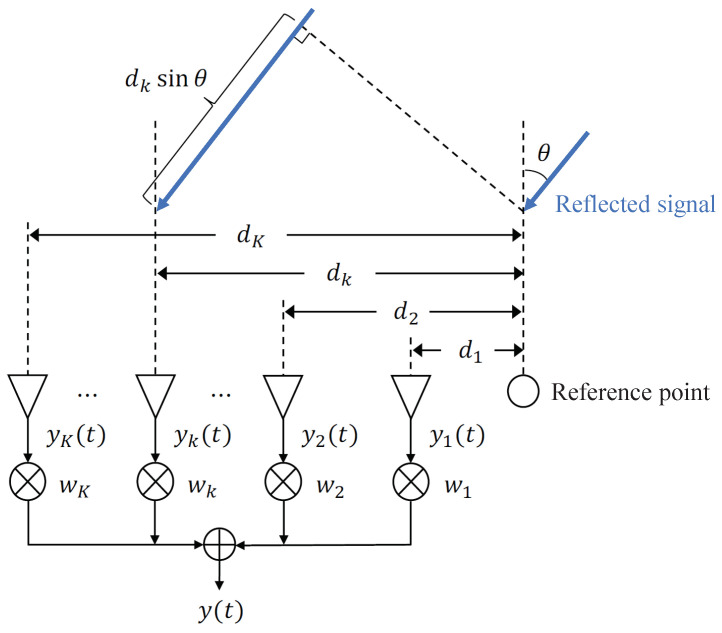
*K* receive antennas arranged linearly.

In the MIMO FMCW radar system with multiple transmitting and receiving antennas arranged linearly, a further phase shift occurs. Figure 2 shows a linear arrangement of *K* receiving antennas. Here, let θ be the angle of incidence of the reflected signal; the received signal at the *k*-th receiver, $y_{k}\left(t\right)$, is expressed by the following equation:

(9) $$\begin{array}{c} y_{k}\left(t\right)=Ae^{j(2\pi f_{b}t+\Phi \left(t\right)+\Delta \phi \left(t\right)+\frac{2\pi }{\lambda }d_{k}\sin\theta )}, \end{array}$$

where λ is the wavelength of the carrier and $d_{k}$ is the relative distance between the receiving antenna and the reference point as shown in Figure 2. Based on the beamforming weight $w_{k}$, the received signal for a specific beam direction is obtained as

(10) $$\begin{array}{c} Y\left(t\right)=\sum_{k=1}^{K}y_{k}\left(t\right)w_{k}. \end{array}$$

From the above explanation, by specifying $R_{\mathrm{const}}$, i.e., the distance between the MIMO FMCW radar and the subject, and the beam direction θ, it is possible to extract the phase change due to the relative displacement of the subject.

## 3. Related Work

This section describes previous research on MIMO FMCW radar-based human detection and localization and radar-based heartbeat detection.

### 3.1. Estimation of Human Position Using MIMO FMCW Radar

For heartbeat detection using MIMO FMCW radar, the location of the subject must be estimated with high accuracy. To estimate the location of an object using MIMO FMCW radar, the conventional methods first calculate a range-angle map that represents the signal power at each position separated by range and angle. On this range angle map, the location where the signal power exceeds the threshold value is detected as the object location. Here, a threshold setting algorithm such as Constant False Alarm Rate (CFAR) [18] is used. However, the threshold value used for human detection depends on the location of the radar and the surrounding environment. Therefore, the detection accuracy of object location may decrease depending on the threshold value. Specifically, locations where the target object does not exist may be detected incorrectly due to noise or multipath components.

To decrease the influence of such clutter noise, K. Han et al. proposed a human detection method based on the Curve-Length (CL) method [19]. By comparing the I/Q trajectory extracted from the position where the human was detected and the wall clutter, they observed that the I/Q trajectory in the former case is circular, corresponding to the body motion of the person, in contrast to the I/Q trajectory of the latter, which changes very little. Therefore, the human position was detected based on the calculated curve length of the I/Q trajectory. This method can detect human positions more accurately than the conventional method [18]. However, if noise and multipath components caused by objects other than humans are large, the accuracy of object location detection degrades.

**Figure 3:**
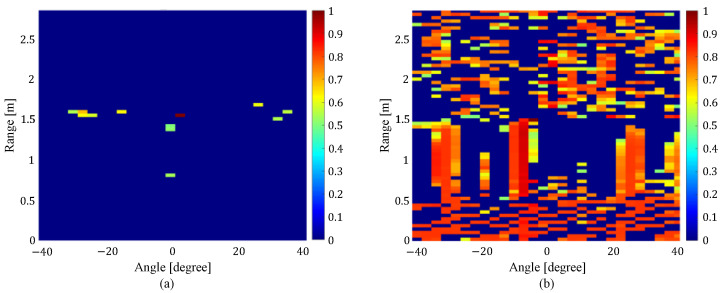
Correlation map between (**a**) the cells corresponding to the human location and all cells on the range-angle map, and (**b**) the cells corresponding to noise and multipath components and all cells on the range-angle map [17].

**Figure 4:**
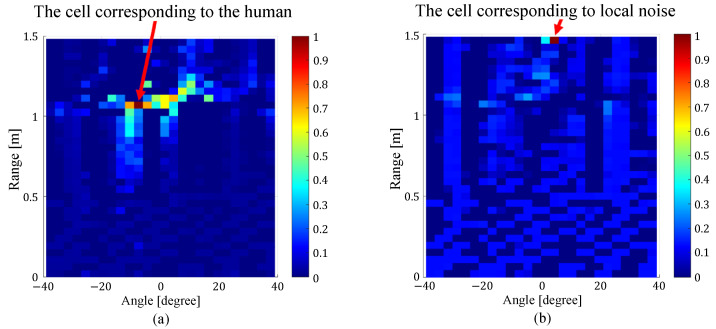
Correlation map between (**a**) the cells corresponding to the human location and all cells on the range-angle map, and (**b**) the cells corresponding to local noise and all cells on the range-angle map [17].

To solve this problem, Endo et al. focused on the correlation of the curve lengths of the received I/Q signals between the cells with the high power and all cells on the range angle map [17]. Here, “cell” refers to a single unit of space when the radar coverage area is divided by a specified angle and distance. As shown in Figure 3, the cells corresponding to the position of a human have high correlation values only around the cell, while the cells corresponding to noise and multipath components have high correlation values in the whole range-angle map. By using the features of the correlation map, the noise and multipath components can be excluded, and the accuracy of human location detection was confirmed to be improved over the CL method [19]. However, when local noise occurs, the number of cells with high correlation values with that cell decreases, which may lead to false detection of the cell as a location where a person is present. Note that “local noise” refers to noise that occurs only in that cell, and not in other cells. To deal with the local noise, the correlation values with the cell’s two neighboring ones were used for human location estimation. In addition, features related to the power in the cells in question and the overall correlation value were used. As shown in Figure 4, the correlation values between the cell corresponding to the local noise and the cells on both sides are smaller than the correlation values between the cell corresponding to the human location and the cells on both sides. By using this feature, it was confirmed that the effect of local noise can be reduced and the accuracy of human location estimation can be improved.

This paper estimates the human location within the radar irradiation range based on the conventional method [17].

### 3.2. Radar-Based Heart Rate Estimation

In daily life, it is preferred to estimate heart rate without the use of wearable devices for monitoring purposes. Therefore, there are many studies on non-contact heart rate estimation using cameras [7] and radar [11,12,14]. In the camera-based heart rate estimation, the ICA-based method [7] has been developed. Since heart rate varies with blood pressure, and hemoglobin in blood has the property of absorbing the green component of light, the heart rate is estimated by observing the reflectance of light. Thus, by performing ICA on the color channels of a face image, it is possible to isolate only the color change due to heartbeat. Based on this fact, Poh et al. [7] proposed a method for estimating heart rate from face images captured by a low-cost webcam. First, ICA is applied to the time waveform of RGB values obtained from the face image to obtain a plethysmogram. Here, plethysmogram means waveform data in which the expansion and contraction of skin blood vessels are captured as waveforms from the skin surface. By extracting the heartbeat component from this waveform data and the heartbeat interval (RRI: R-R interval), a numerical value of the heart rate can be obtained. However, the drawbacks of the camera-based method are the privacy issues and the degradation of detection accuracy in dark environments.

In contrast, the radar-based heartbeat detection method has the advantages of high privacy and the ability to detect heartbeats while wearing clothing. Therefore, heart rate estimation using Doppler radar [11,12] and FMCW radar [14] has been actively studied. Doppler radar transmits microwaves toward an object and analyzes the reflected Doppler-shifted microwaves to determine the target’s velocity and direction. However, accurate heartbeat detection is difficult because the power of the heartbeat component to the Doppler sensor’s reflected signal is lower than the power of respiration or body motion. Generally, displacement of the chest due to heartbeat is 0.2 mm to 0.5 mm, and displacement due to respiration is 4 mm to 12 mm [20]. In addition, displacement due to body motion is often greater than displacement due to respiration or heartbeat, and such body motion can significantly reduce the accuracy of heartbeat detection. To extract heartbeat components from signals containing not only heartbeat components, but also non heartbeat components such as respiration and body motion, various Doppler radar-based heartbeat detection methods have been proposed [11,12,14].

In general, the normal respiratory rate varies between 0.1 and 0.3 Hz, and the normal HR is between 0.5 and 2.0 Hz. Based on this fact, Yamamoto et al. [11] proposed a spectrogram-based heart rate estimation. In this method, a spectrogram is calculated from the received signal of the Doppler radar, and the spectrum corresponding to a heartbeat is integrated from the spectrogram. The RRI is then estimated by using the peaks before and after the peak to be investigated from the peaks of the integrated spectrum to detect the peaks caused by heartbeats. One issue with this study is that it is difficult to extract the heartbeat frequency component when the respiration and heartbeat frequencies are close or when the respiration and noise components are larger than the heartbeat component.

On the other hand, Kitagawa et al. [12] proposed a heart rate estimation method based on the Viterbi algorithm. In the conventional method [12], a multi-beam Doppler radar is used. First, integrated values of the spectrum are calculated using received signals from multiple beam directions to detect peaks. Next, multiple peaks occurring within 0.1 s are considered as a single peak candidate, and the average time at which each peak occurs is calculated as the peak candidate occurrence time. Finally, the Viterbi algorithm is used to detect the peak set that is most likely to be caused by a heartbeat from the peak candidates. One issue with this study is that if the time window used for heartbeat estimation is short, the algorithm may be unreliable due to insufficient statistical features.

In contrast, the heart rate estimation method using FMCW radar [14] estimates heart rate based on phase variation due to relative chest displacement caused by heartbeats. Zheng et al. [14] propose heart rate estimation using reconstructed signals based on VMD. VMD is based on the variational principle and decomposes the signal into optimized frequency modes by a combination of center frequency and bandwidth. This makes it possible to analyze and extract signals in specific frequency bands. Based on this fact, in the conventional method [14], the chest wall signal is decomposed into multiple frequency modes using VMD, and the IMF signal belonging to each mode is extracted. The IMF signal whose center frequency is within the frequency range of the second harmonic signal of the heartbeat is then selected. The range of the second harmonic frequency of the heartbeat is between 2.0 and 4.0 Hz. The heart rate is then estimated by detecting frequency peaks from the reconstructed signal generated by weighted addition based on the energy of each IMF signal. The first issue with this study [14] is the use of the SISO FMCW radar, which may degrade the estimation accuracy due to components reflected from objects other than humans. In addition, second harmonic signals have smaller amplitudes than the fundamental and are more difficult to extract. Nonetheless, since the estimated heart rates before and after are not taken into account, the variation in the estimated heart rates in adjacent time windows may be excessively large. Furthermore, these methods [11,12,14] are effective in situations where the subject is directly in front of the Doppler and FMCW radars, but not in situations where the subject is not directly in front of the Doppler and FMCW radars due to antenna directivity. In contrast, MIMO FMCW radar can estimate the DoA and receive microwaves from the estimated DoA using beamforming techniques. In [21], Bouazizi et al. proposed a system that uses a MIMO CW Doppler radar and 3D Light Detection and Ranging (LiDAR) to locate the subjects and estimate their heart rate. Using the 3D LiDAR, they were able to identify the chest location of the subjects, and by applying receive beamforming with the MIMO CW Doppler radar, they were able to capture the signals related to the heartbeat. However, their reliance on a second device for the localization of the subjects makes their method less practical. Such a device is not needed when an FMCW radar is used, as the FMCW can identify the subject and apply beamforming to capture their heartbeat signals.

Arsalan et al. [22] presented a Kalman filter-based method for contactless and noninvasive monitoring of respiration and heart rate using radar systems. Their approach improves heart rate stability and accuracy by successively narrowing the bandwidth of a band-pass filter and updating filter limits based on the target’s current heart rate. The algorithm effectively ignores measurement segments affected by random body movements. Validation with a 60-GHz FMCW radar system demonstrated that their method provided accurate heart rate measurements, which were compared with data from a commercial chest belt across various breathing conditions in 14 subjects.

Huang et al. [23] developed a real-time heart rate detection method using 77 GHz FMCW radar. Their approach establishes a new motion model to extract chest and abdominal motion signals, effectively eliminating random body motion (RBM) through a combination of polynomial fitting and recursive least squares (RLS) adaptive filtering. Additionally, they employed multi-detection-point adaptive harmonics cancellation (AHC) to remove respiratory harmonics. The introduction of a spectrum analysis algorithm based on linear predictive coding (LPC) further enhances accuracy. Experimental results indicated that their method significantly reduced RBM and respiratory harmonics, achieving an average heart rate detection error rate of just 2.925%.

Jung et al. [24] introduced a novel method for reducing measurement time in remote heart rate detection using millimeter-wave FMCW radar. Their approach utilizes sampling theory and the frame structure of FMCW radar systems, allowing for improved resolution of heart rate measurements within just 2.5 s by adopting multiple sampling rates. Evaluations with a commercial FMCW radar system showed that their method achieved lower measurement errors and shorter measurement times compared to traditional ECG sensors and conventional signal processing techniques.

Hu and Toda [25] proposed a method for radar-based vital sign monitoring that accommodates moving targets using millimeter-wave FMCW radar. Unlike previous studies that required subjects to remain stationary, their approach allows for accurate heart rate detection even when subjects are walking or facing different directions. Experimental results demonstrated a heart rate estimation accuracy of 95.88%, with an RMSE of 4.09 bpm during fixed-route tests, showcasing the method’s potential for non-contact monitoring in dynamic scenarios.

Zhou et al. [26] presented an advanced processing scheme for improving non-contact heart rate detection using mm-Wave radar. The method involves adaptive range bin selection based on variance to target accurate phase information, followed by smooth spline fitting to purify the phase signal. Matched filtering is then used to mitigate noise and interferences like breathing and random body movements. The heartbeat signal is further refined using Variable Mode Extraction (VME), and a novel Double-Chirp Z-Transform (Double-CZT) technique is employed for precise frequency measurement. Experimental results showed a mean absolute error of less than 1 bpm, indicating enhanced accuracy and signal-to-noise ratio.

More recently, Zhou et al. [27] investigated noncontact heart rate detection using FMCW radar, addressing the impact of respiratory harmonics. They proposed a method incorporating singular spectrum analysis (SSA) to enhance harmonic components, achieving an HR detection accuracy of 98.51% with an RMSE of 1.98 BPM.

Kim et al. [28] proposed a method for accurate heart rate estimation using FMCW radar combined with a lightweight deep learning model called HeartBeatNet. Their approach involves unique signal processing techniques to capture respiratory and heart rate characteristics, enabling effective training of the network. The experimental results indicate that their method achieves high accuracy in heart rate estimation in a short time, making it suitable for real-world industrial applications due to its low computational cost.

An early version of this work was submitted for publication [29]. In this work, we estimate the HR by firstly identifying the human location and use the signal reflected from identified location for HR estimation. In [29], we only consider the scenario of a single subject, we run limited evaluations and did not compare the results of estimation using FMCW radar to those using Doppler radar.

Based on the above research, this paper aims to reduce the influence of factors other than heartbeats by using VMD to extract heartbeat components from the received signal. In addition, by using the MIMO FMCW to estimate the two-dimensional position of a human including distance and angle information, the influence of reflected signals from objects other than humans will be reduced. Furthermore, after selecting a reliable reconstructed signal by signal quality assessment, this paper aims to reduce excessive variations in the estimated value by selecting an appropriate estimated heart rate from the estimated values of multiple cells by considering the time transition of the estimated heart rate.

## 4. Proposed Method

**Figure 5:**
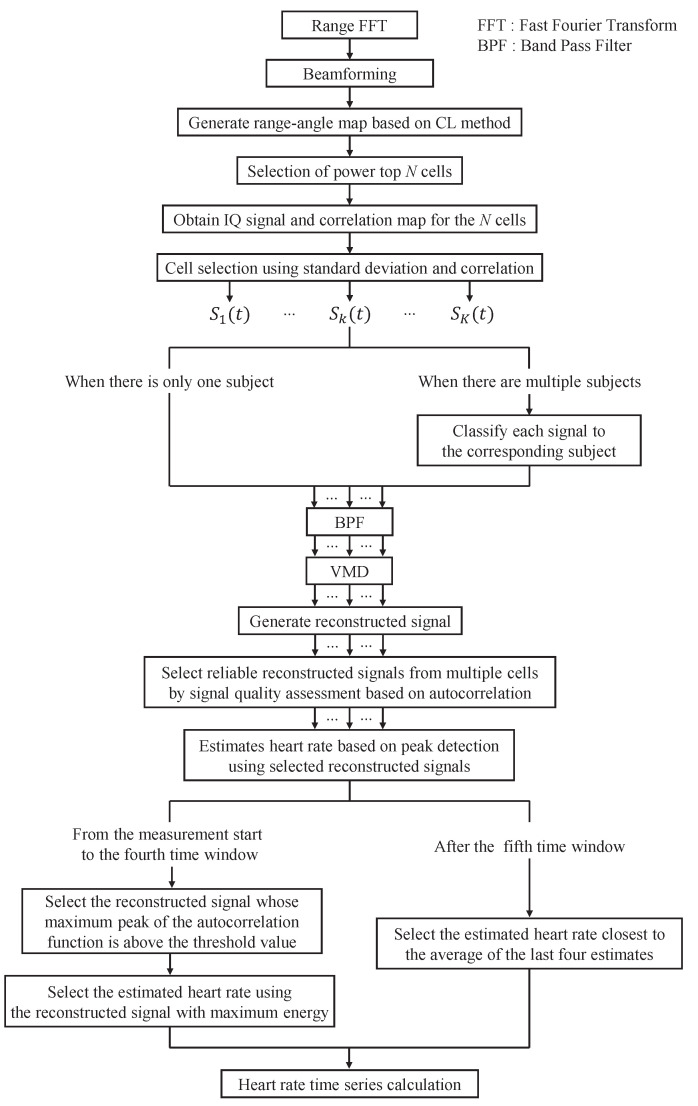
A flowchart of the proposed MIMO FMCW radar-based heartbeat detection method.

In this section, we describe in detail our method for heart rate estimation considering reconstructed signal features based on VMD via MIMO FMCW Radar. The proposed method is divided into two parts: (i) estimation of human position, and (ii) heart rate estimation considering features of the reconstructed signal based on VMD. The flowchart of the proposed method is shown in Figure 5. In the following, we first explain the method for estimating the position of a person, followed by the heart rate estimation method.

### 4.1. Estimation of Human Position

**Figure 6:**
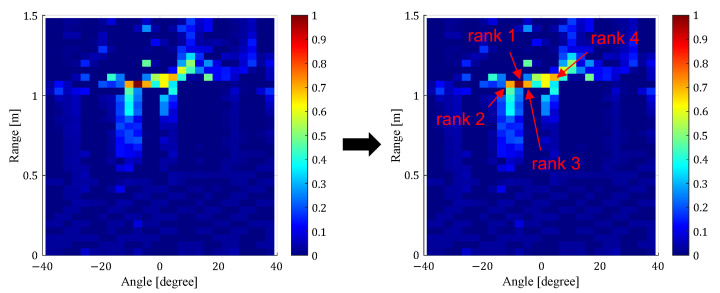
An example of the detection of the top power *N* cells.

In the human detection and localization step, the position of a human is estimated from the radar irradiation range based on previous studies [17]. First, the range Fast Fourier Transform (FFT) is applied to the received signal Y(t) from each beam direction in 2 s steps for each 20 s time window to calculate the distance to the object. The length of the time window and the step length were set empirically. Next, beamforming is used to obtain IQ signals for each specified angular range. A range angle map is then generated based on the CL method [19], with the power being the curve length of each IQ signal. The CL method calculates the length of variation of the IQ signal on the IQ plane within the Coherent Processing Interval (CPI) at every specified range and angle as follows:

(11) $$\begin{array}{c} CL=\sqrt{\sum_{\tau =2}^{T_{M}}({\left(I[\tau ]-I[\tau -1]\right)}^{2}+{\left(Q[\tau ]-Q[\tau -1]\right)}^{2})}, \end{array}$$

where $T_{M}$ is a CPI, I[τ] and Q[τ] are the in-phase and quadrature signals on a specified range, angle, and τ is a sampling time, respectively. Next, as shown in Figure 6, the top *N* cells with the highest power on the range angle map calculated by the CL method are detected as candidates for cells with human presence. For each of the *N*-detected cells, the correlation values of the received I/Q signals with all the cells on the range angle map are calculated. To emphasize human-induced cells, only cells with a correlation value equal to or higher than 0.5 are extracted, and a correlation map of the *N*-detected cell points is calculated. The cells to be used for heart rate estimation are then selected based on the correlation values and standard deviation [17]. The standard deviation of the correlation value with each cell of the correlation map is first calculated. As a result, it was confirmed that the standard deviations of the cells corresponding to noise and multipath components are as high as 0.2 to 0.5, while the standard deviations of the cells corresponding to human positions are 0.02 to 0.10. To reduce the effect of local noise, the maximum correlation value between each cell and both neighboring cells is also calculated. As shown in Figure 4, the correlation values between the cell corresponding to the person’s position and the cells on both sides are as high as 0.4 to 0.7, while the correlation values between the cell corresponding to local noise and both neighboring cells are as small as 0.1 to 0.3. Based on the above, among the top *N* cells, all cells that satisfy both a standard deviation of 0.2 or less and a maximum correlation value of 0.4 or more with their respective two neighboring cells are selected as cells corresponding to the human position. In this study, each threshold value was selected empirically. If there is only one subject, the received signals in the selected cells are used to estimate the heart rate. If there are multiple subjects, each selected cell is classified as to which subject it corresponds to. Specifically, the distance and angle between cells selected as being caused by human are compared, and cells are classified as being caused by another subject if they are more than a specified distance or angle away from each other. Here, the specified distance and angle are set to 20 cm and 6°, respectively. These values are set empirically based on the fact that the size of one cell is 4 cm in distance and 3° in angle.

### 4.2. Heart Rate Estimation Based on Features of the Reconstructed Signal

First, a Band-Pass Filter (BPF) is applied to the I/Q signal in each cell selected based on the standard deviation and correlation values in the correlation map. The cutoff frequency is set to 0.8–2.0 Hz, which corresponds to heart rates ranging from 48 to 120 bpm. This range encompasses the normal heart rate frequency for adults, which is generally between 60 and 100 bpm, while also accounting for higher rates that may occur during light physical activity and lower rates that may occur for elderly people and that come with age. The chosen frequency range ensures that the proposed method remains effective for practical applications in daily heart rate monitoring. Next, the BPF-applied signal is decomposed into multiple IMF signals based on VMD. Here, the number of decompositions is set to 10, as concluded from the results of our preliminary experiments. Based on the energy of each decomposed IMF signal, the reconstructed signal is generated by weighting. Here, among the generated IMF signals, the IMF signals whose center frequency is within the normal heart rate frequency range are selected. The energy of this selected IMF signal is calculated and the reconstructed signal W(t) is generated based on its value as shown in Equation (12).

(12) $$\begin{array}{c} W\left(t\right)=\frac{e_{1}}{e_{1}+\cdots +e_{L}}S_{1}\left(t\right)+\cdots +\frac{e_{L}}{e_{1}+\cdots +e_{L}}S_{L}\left(t\right). \end{array}$$

**Figure 7:**
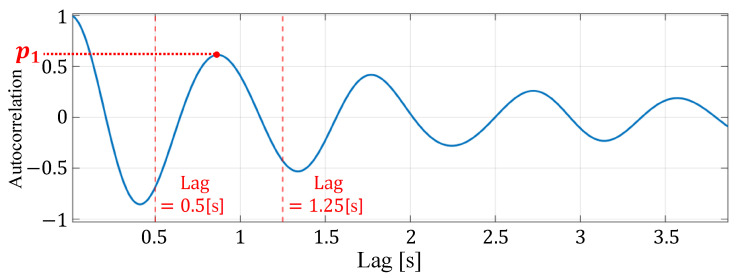
An example of peak detection of the autocorrelation function of the reconstructed signal.

Here, *L* is the number of IMF signals selected, [$S_{1},S_{2},\cdots ,S_{L}$] is each IMF signal, and [$e_{1},e_{2},\cdots ,e_{L}$] is the energy of each IMF signal. The energy of the signal is calculated from the sum of squares of the amplitudes of each IMF signal. The signal quality assessment based on the autocorrelation value is then used to select a highly reliable reconstructed signal from the reconstructed signals of multiple cells, aiming to reduce the influence of factors other than heartbeats. The autocorrelation function of the reconstructed signal is calculated as shown in Figure 7, and peaks contained within a time interval corresponding to a normal heartbeat interval are detected. The normal heartbeat interval is 0.50 to 1.25 s. If the number of peaks detected here is one, or if the ratio of the amplitude of the second largest peak to the largest peak is less than the threshold $th_{r}$, as shown by Equation (13), the signal is considered a reliable signal due to heartbeat and the reconstructed signal is used for heart rate estimation.

(13) $$\begin{array}{c} \frac{p_{2}}{p_{1}}\leq th_{r}. \end{array}$$

Here, $p_{1}$ represents the autocorrelation value at the peak with the highest amplitude, and $p_{2}$ represents the autocorrelation value at the peak with the second highest amplitude. The threshold value $th_{r}$ is set to 0.6 as observed from the results of our preliminary experiments. Note that our preliminary experiments follow the same experimental settings used in this work. They have been used to identify the number of decompositions and the threshold $th_{r}$. We evaluated different values of both parameters to identify their optimal values. The scenarios used in the preliminary experiments have been discarded afterwards and are not part of the scenarios used for evaluation.

In cases other than the above, the corresponding reconstructed signal is excluded as unreliable. If the reconstructed signals in all cells are excluded, the time window is excluded from estimation. The heart rate is then estimated in each cell based on peak detection using the reconstructed signals that were not excluded in the previous procedure.

Finally, the appropriate estimated heart rate is selected from the estimated heart rates in each cell based on time transition. From the start of the measurement to the fourth time window, the autocorrelation function of each reconstructed signal is first calculated, and the signal whose maximum peak exceeds the threshold value $th_{c}$ is selected. The threshold $th_{c}$ is empirically set at 0.55. This aims to extract reconstructed signals with strong periodicity from the reconstructed signals in multiple cells. Next, the energy of each reconstructed signal was calculated and the error between the estimated heart rate from those signals and the true heart rate was compared. Table 1 shows the energy of each reconstructed signal and the error between the heart rate estimated from those signals and the true heart rate. As a result, we confirmed that the error in the estimated value by the reconstructed signal with the maximum energy was the smallest, as Table 1 shows. Thus, the estimated heart rate using the reconstructed signal with the maximum energy is selected. The number of these time windows was empirically set to four. After the fifth time window, the estimated heart rate that is closest to the average of the last four estimated heart rates is selected. By selecting an appropriate estimate from the estimated heart rates in multiple cells in this way, considering the energy of each reconstructed signal or the time transition of the heart rate, the aim is to reduce the extraneous variation in the estimates.

## 5. Experimental Evaluation

To evaluate the accuracy of the heart rate estimation of the proposed method, we conducted experiments using Doppler radar and MIMO FMCW radar to observe the heart rate. In this section, we first describe the experimental setup and then explain the heart rate estimation results of the proposed method.

### 5.1. Experimental Setup

To evaluate the estimation accuracy of the proposed method, we conducted experiments to observe the heart rate. The specifications of the used Doppler radar and the used MIMO FMCW radar are listed in Table 2 and Table 3, respectively. The carrier frequency of the Doppler radar and the MIMO FMCW radar were 24 GHz and 79 GHz, respectively, and the sampling frequencies of the radars were set as 1000 Hz and 240 Hz, respectively. The parameters used in the experiments are also listed in Table 4.

Alongside the Doppler/FMCW radars, an ECG device has been attached to the chests of the subjects to collect their heartbeat signal. The device is manufactured by BioSignalPlux [30]. The Radar data are synchronized with the ECG ones which we use as ground truth for evaluation.

**Figure 8:**
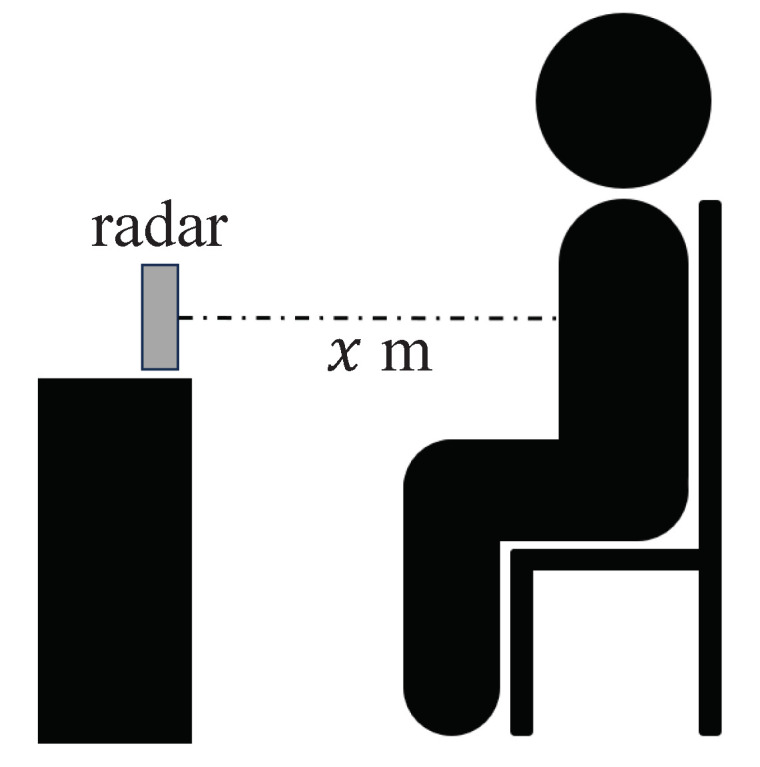
The experimental environments for case (i).

**Figure 9:**
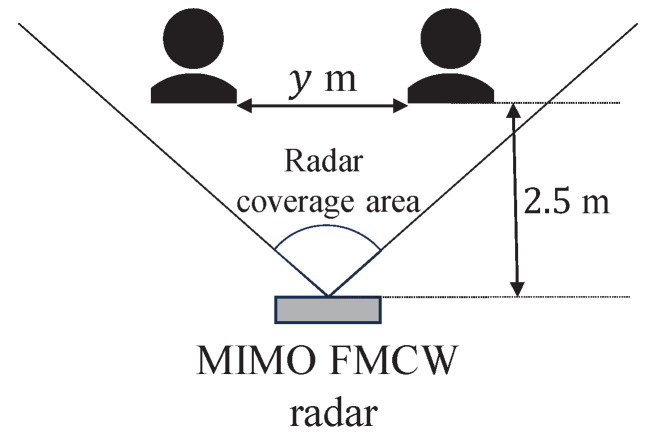
The experimental environments for case (ii).

As for the experimental settings, we assumed two cases: (i) only one subject in the radar coverage area, and (ii) two subjects in the radar coverage area as shown in Figure 8 and Figure 9, respectively. In case (i), the distances between the subject and the radar were 1.0 m and 2.0 m. In case (ii), the distance between the subject and the radar was set to 2.5 m, and the distance between the two subjects was set to 0.5 m and 1.0 m. The number of subjects was four for case (i) and two for case (ii). The observation time was 2 min for case (i) and 1 min for case (ii).

Also, the threshold for the ratio of the second peak to the largest peak in the autocorrelation $th_{r}$ was set to 0.6 empirically. The ground truth value is calculated based on the data observed by ECG. In this experiment, the angles considered for the beamforming range of MIMO FMCW radar are from −40° to 40°. The beamforming interval was also set to 3°, which means 27 angular beams were acquired. Furthermore, the number of selected cells *N* by the CL method was empirically set to 10 for case (i) and 10×numberofsubjects for case (ii). However, the number of subjects was assumed to be known.

As the performance metric, the Mean Absolute Error (MAE) between the estimated heart rate and the ground truth one is used, and is calculated as Equation (14).

(14) $$\begin{array}{c} \mathrm{MAE}=\frac{1}{P_{\mathrm{est}}}\sum_{i=1}^{P_{\mathrm{est}}}\left|HR_{\mathrm{est}}\left(i\right)-HR_{\mathrm{ref}}\left(i\right)\right|, \end{array}$$

where $P_{\mathrm{est}}$ denotes the number of nonzero estimated heartbeats, $HR_{\mathrm{est}}\left(i\right)$ and $HR_{\mathrm{ref}}\left(i\right)$ denote the *i*-th estimated heart rate and the ground truth one, respectively. The exclusion rate of heartbeat detection is then calculated as Equation (15).

(15) $$\begin{array}{c} \mathrm{Exclusion}\;\mathrm{rate}\;\left[\%\right]=\frac{P_{\mathrm{zero}}}{P_{\mathrm{all}}}\times 100, \end{array}$$

where $P_{\mathrm{zero}}$ and $P_{\mathrm{all}}$ denote the number of zeros in the estimates and the number of total estimated heartbeats, respectively.

### 5.2. Results for the Heart Rate Estimation

We conducted the experiment in two cases: (i) when there was only one subject within the radar coverage area, and (ii) when there were two subjects within the radar coverage area. The results of heart rate estimation in the two cases are described below, respectively.

#### 5.2.1. Heart Rate Estimation Results for Case (i)

In case (i), one subject was placed within the radar coverage area to observe heartbeats. First, Table 5 shows a comparison of MAE for heartbeat estimation using the proposed method and methods based on the three related studies presented in Section 3 via MIMO FMCW radar. To compare the radars used for the measurements, Table 6 shows the estimation results when using Doppler radar for each method. When using MIMO FMCW radar, the cell corresponding to a human location was estimated based on the CL method [17], and then the signals in that cell were used to estimate the heart rate for each method. Once again, we describe the three related studies for comparison. In the spectrogram-based method [11], a spectrogram is calculated based on the received data, and the peak is detected from the spectral integral value caused by the heartbeat. In the Viterbi algorithm-based method [12], the average of multiple peaks in the spectral integrals is used as a peak candidate, and the most reliable set of peaks attributable to a heartbeat is selected based on the Viterbi algorithm. The VMD-based method [14] detects peaks by generating a reconstructed signal using IMF signals whose center frequency is within the frequency range of a heartbeat. In these three methods, when MIMO FMCW radar is used, the average value of the estimated heart rate in multiple cells is calculated as the estimated value. In contrast, the proposed method selects a reconstructed signal for heart rate estimation from the reconstructed signals in multiple cells by signal quality assessment based on autocorrelation values. The signal quality assessment selects a reconstructed signal as reliable if it has one peak in the normal heartbeat interval in the autocorrelation function of the reconstructed signal, or if the ratio of the second peak to the largest peak is greater than a threshold value. Furthermore, the estimated heart rate closest to the average of the last four estimated heart rates among the estimated heart rates in multiple cells was selected.

Table 5 and Table 6 show that the MAE is smaller when using MIMO FMCW radar than Doppler radar for all the methods. This is because, unlike Doppler radar, when using MIMO FMCW radar, the location of the person is estimated in advance and only the signals at that location are extracted for heartbeat estimation, thereby reducing the influence of components reflected from objects other than the human. It can be also seen that the MAE is the smallest when MIMO FMCW radar is used with the proposed method among all the methods. This result will be discussed later.

**Figure 10:**
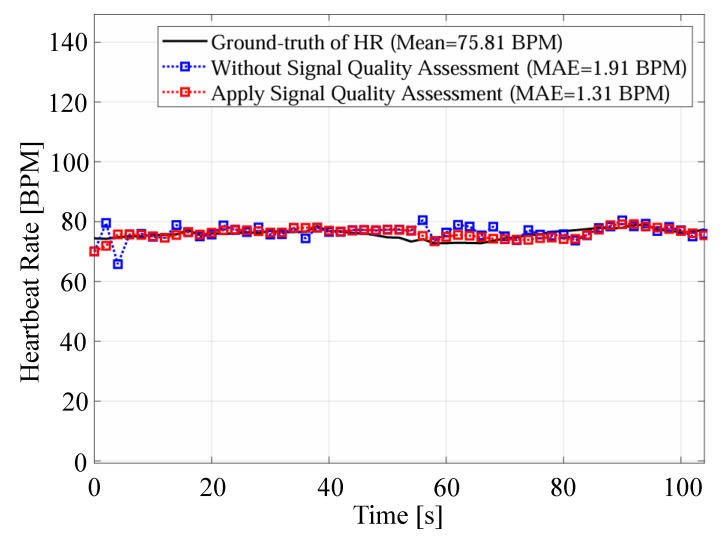
An example of the comparison of estimated heart rates with and without signal quality assessment.

**Figure 11:**
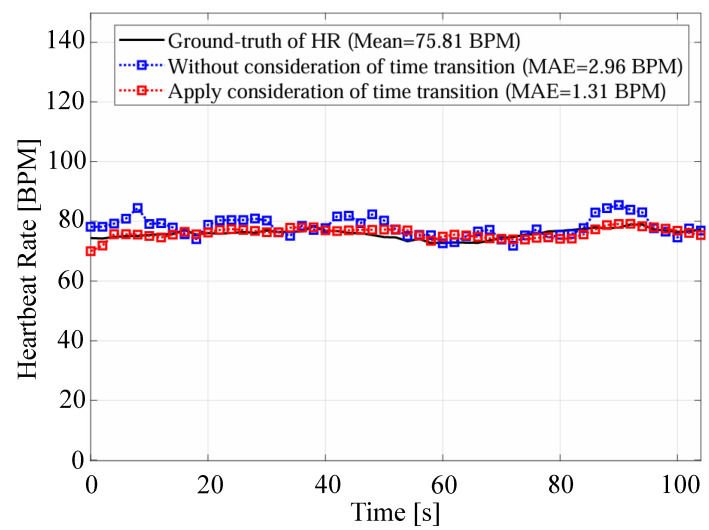
An example of the comparison of estimated heart rates with and without time transition consideration.

First, we compare the proposed method with the methods based on the spectrogram [11] and the Viterbi algorithm [12]. In the proposed method, the reconstructed signal based on VMD is used for heartbeat estimation, which could reduce the influence of components caused by other than the heartbeat. Next, we compare the proposed method with the conventional method based on VMD [14]. In the proposed method, unlike the conventional method based on VMD, the reconstructed signal used for heart rate estimation is selected based on signal quality assessment. In addition, the estimated heart rate closest to the average of the four most recent estimates was selected from the estimated heart rates in multiple cells by considering the time transition. To demonstrate this effect, Figure 10 and Figure 11 present examples comparing estimated heart rates under different conditions: with and without signal quality assessment, and with and without consideration of time transition, respectively. In Figure 10, it is clear that excluding unreliable signals through signal quality assessment allows the proposed method to achieve estimates closer to the true heart rate. Furthermore, Table 7 reports the average variation in true heart rate between neighboring time windows, as calculated from the ECG data. The mean variation across all time windows and subjects is 0.76 bpm, indicating relatively stable heart rate fluctuations. Moreover, Figure 11 shows that considering the time transition in the estimation process effectively reduces excessive variation in the estimated heart rate, thereby improving accuracy. This approach smooths rapid changes in the estimated heart rate, leading to a smaller overall estimation error. The combined results from Table 7 and Figure 10 and Figure 11 demonstrate that incorporating signal quality assessment and time transition considerations improves the accuracy and reliability of the proposed heart rate estimation method.

**Figure 12:**
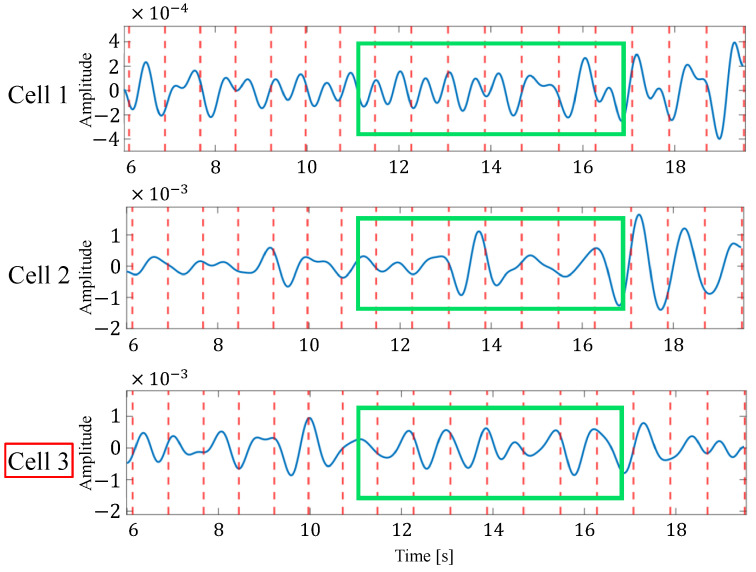
An example of the list of each reconstructed signal when selecting an appropriate signal from the reconstructed signals generated in multiple cells. Here, the green box indicates the time window in which the observation is made.

Figure 12 shows an example of the list of each reconstructed signal when selecting an appropriate signal from the reconstructed signals generated in multiple cells by considering the time transition. In this figure, the solid blue line represents the reconstructed signal and the red dotted line represents the actual heartbeat generation position calculated from the ECG. In the example in this figure, the third cell is selected as the appropriate signal. From this figure, it can be seen that some reconstructed signals have peaks close to the actual heartbeat, while others have many extraneous peaks, such as the signal in the first cell, and others have no clear peaks, such as the signal in the second cell. Therefore, the proposed method could improve the accuracy of heartbeat estimation by selecting the appropriate reconstructed signal out of these signals based on the energy or the most recent estimate.

Next, we compare the MAE reported for the different methods when Doppler radar is used. Table 6 shows the MAE of estimated heart rate using Doppler radar. From Table 6, it can be seen that the proposed method has multiple data with a larger MAE than the conventional method based on the spectrogram and Viterbi algorithm. When using Doppler radar, since the position of a human is not estimated, signals that include components reflected from non-human sources are used for heartbeat estimation. For this reason, when the signal is decomposed into multiple components using VMD, the components reflected from non-human sources can be omitted, but the heartbeat component cannot be separated from the human-induced components such as respiration and body motion, which is thought to have degraded the estimation accuracy of the proposed method. Next, Table 8 shows a comparison of the exclusion rate for the proposed method using the MIMO FMCW radar and the Doppler radar. From Table 8, it can be seen that the proposed method using the MIMO FMCW radar results in a lower exclusion rate than the case using the Doppler radar. As mentioned earlier, by using the MIMO FMCW radar, only signals caused by humans are extracted in advance. Furthermore, when using the MIMO FMCW radar, signals in multiple cells selected as human-induced are used for heartbeat estimation. Therefore, the reconstructed signals generated by the MIMO FMCW radar are more reliable than those generated by the Doppler radar, and the number of signals excluded from the signal quality assessment is considered to be low. This led to a reduction in the exclusion rate.

**Figure 13:**
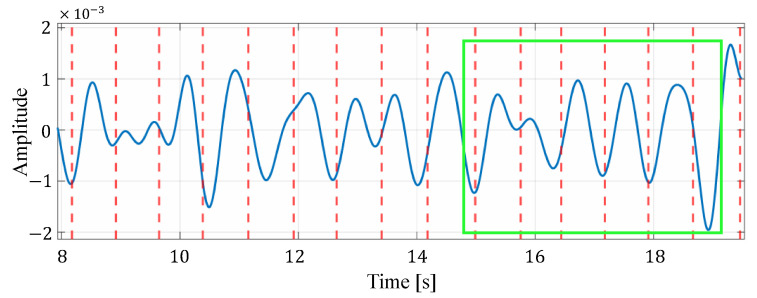
An example of a reconstructed signal selected based on energy only in case (i).

**Figure 14:**
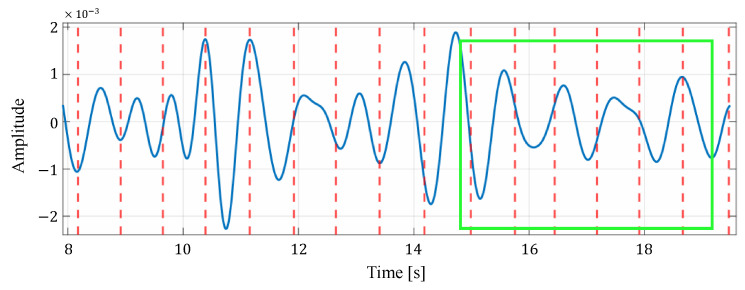
An example of a reconstructed signal selected based on energy and periodicity in case (i).

Furthermore, in selecting the appropriate estimate from the estimated heart rates of multiple cells in the proposed method, we compare the case where the selection is based on only energy with the case where the selection is based on energy and periodicity. Table 9 shows the heart rate estimation results for both cases. From Table 9, it can be seen that for four data, the MAE is smaller when both energy and periodicity are considered than when only energy is considered. This is because the reconstructed signal with strong periodicity was selected so that a signal with a clear peak due to the heartbeat could be used for the heartbeat estimation. Figure 13 and Figure 14 show an example of a reconstructed signal selected based on energy only and a reconsidered painter signal selected based on energy and periodicity, respectively. Figure 13 shows that the amplitude of the signal is not as constant as the signal in Figure 14, but the peak occurs relatively close to the heartbeat position of the true value. This indicates that, in the reconstructed signal selected, based on the energy and periodicity that Figure 14 shows, a small component attributable to the human heartbeat has been excluded from the estimation. For this reason, we consider that, in case (i), the MAE was degraded in some data by considering both the periodicity and energy of the reconstructed signal.

#### 5.2.2. Heart Rate Estimation Results for Case (ii)

In case (ii), two subjects were placed within the radar coverage area to observe heartbeats. The distance between the radar and the subjects was 2.5 m, and the distance between the two subjects was 0.5 m and 1.0 m. The results of heart rate estimation by the three conventional methods [11,12,14] and the proposed method are shown in Table 10. Table 10 shows that the proposed method has smaller MAE than the methods based on spectrogram, Viterbi algorithm, and VMD. In case (ii), the signals reflected from different persons are mixed because there are multiple subjects within the radar coverage area. Therefore, when extracting the heartbeat signal of the subject to be estimated, the heartbeat components of different subjects may be included. In the proposed method, MAE could be improved by reconstructing the heartbeat signal based on VMD and then selecting the appropriate signal as the heartbeat signal of the examinee to be estimated based on energy and periodicity when selecting the estimated values up to the fourth time window from the start of measurement.

**Figure 15:**
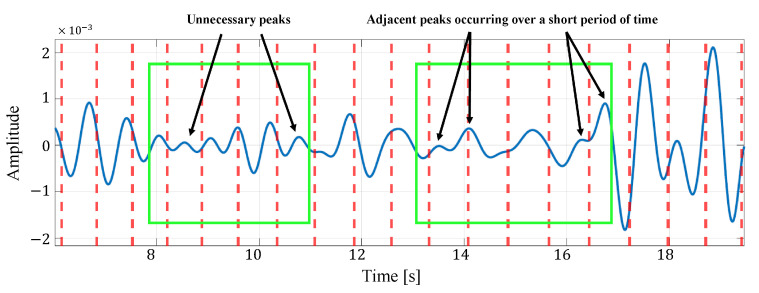
An example of a reconstructed signal selected based on energy only in case (ii).

**Figure 16:**
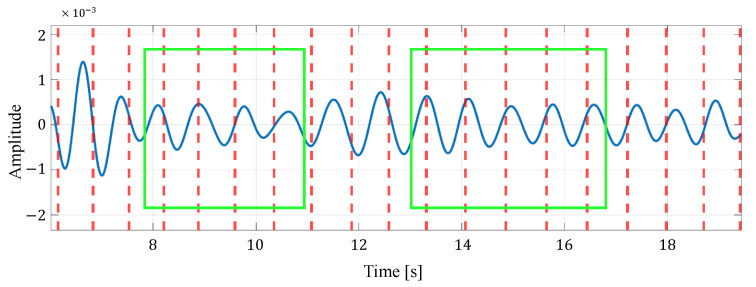
An example of a reconstructed signal selected based on energy and periodicity in case (ii).

Furthermore, in selecting the appropriate estimate from the estimated heart rates of multiple cells in the proposed method, we compare the case where the selection is based on only energy with the case where the selection is based on energy and periodicity. From Table 11, it can be seen that for the three data sets, the MAE is smaller when both energy and periodicity are considered than when only energy is considered. To discuss this factor, Figure 15 shows an example of the reconstructed signal selected when only energy is considered, and Figure 16 shows an example of the reconstructed signal selected when energy and periodicity are considered. In Figure 15 and Figure 16, it can be seen that when energy and periodicity are considered, the peaks occur close to the heartbeat position of the true value, whereas when only energy is considered, multiple peaks occur at different locations from the heartbeat position of the true value. This is because there are multiple subjects within the radar coverage area, and the heartbeat components of different subjects are included in the reconstructed signal. By selecting the reconstructed signal whose maximum peak of the autocorrelation function is above the threshold value, the reconstructed signal with strong periodicity, i.e., the reconstructed signal with small influence of the heartbeat component of the subject who is not the target of estimation, can be used for heartbeat estimation. This led to an improvement in MAE.

However, when classifying the received signals of the radar to which each subject corresponds, threshold values are set for the distance and angle between the subjects to determine them. In this case, if the distance between the subjects is extremely close, it is not easy to classify the received signals. Furthermore, the proposed method assumes that the number of subjects is known. However, it is desirable to estimate the heartbeat without the number of subjects being known. From the above, it is necessary in the future to classify the received signals as reflected signals from each subject based on the correlation value for each signal, and then estimate the heart rate.

That said, our proposed method presents better results than the conventional methods [11,14]. This can be attributed to several reasons. First, of all, the method proposed in [11] relies heavily on the generated spectrograms and their quality. These spectrograms are extracted for the frequency range [3 Hz, 24 Hz], a range that is more susceptible to noise compared to the actual heartbeat range. Nevertheless, this approach is more suited for CW Doppler radar-generated signals: a single signal is used as input from which the spectrogram is generated given the subject’s location is known. When the location is unknown, no particular criterion is set to identify which beams to use. To address this issue, the authors of [12] used all reflected beams, with no particular pre-selection and claimed that this will increase the Signal-to-Noise Ratio (SNR). This might be correct when the subject is less than 20 cm away from the radar, but when the distance is much bigger, the extra reflected beams contain only noise, thus, degrade the SNR. In our work, we rely on data collected from multiple cells, we first identify the location of the subject(s), identify cells that contain useful information and use beams coming from these cells. Not only does this allow our method to narrow down the spatial search space, but it also reduces the amount of noise injected by not accounting for beams coming from other directions. In addition, given that we do not rely on the high frequency ranges, noise, which easily affect these frequencies, the signal used for HR estimation is less perturbed. The VMD-based method [14], on the other hand, detects peaks by generating reconstructed signals using IMF signals whose center frequency is within the frequency range of a heartbeat. However, there is no guarantee that the signals in that frequency range all correspond to the chest movement induced by the heartbeats which leads to wrong detection. To mitigate this problem, in our work, we undergo signal processing that minimizes the effect of other sources of signals within that range, we rely on the periodicity of heartbeats and consider signals from different cells.

### 5.3. Future Work Direction

In the current work, we have shown the potential of CW Doppler radars and MIMO FMCW radars in localizing people and capturing faint chest movements induced by the heartbeats. That said, more recent works have shown potentials of other sensors, such as MIMO-Orthogonal Frequency Division Multiplexing (OFDM). MIMO-OFDM requires a more complex signal processing chain at both the transmitter and the receiver, but provides greater flexibility in managing data transmission across time and frequency domains. This allows for enhanced control over resource allocation and adaptability to varying channel conditions.

In [31], Zhang et al. proposed a framework for integrating target sensing into the channel estimation process for THz massive MIMO (mMIMO) systems with integrated sensing and communication (ISAC). They leverage the sparse nature of THz mMIMO channels to connect channel and target parameters in angular, delay, and Doppler dimensions. By formulating both tasks as structured tensor decomposition problems, they develop a shared channel training pattern to efficiently estimate target and channel parameters. Their tensor-based algorithm extracts key parameters like angles, delays, and Doppler shifts while minimizing training overhead. Simulations show the algorithm performs close to the Cramér–Rao bound. In [32], Lin et al. proposed a tensor-based method for channel estimation and indoor positioning in wideband multiuser millimeter-wave systems. By leveraging sparse channel characteristics and using tensor factorization, they recover key multipath parameters for accurate estimation. They also introduce a clustering-based approach to differentiate multiuser signals, improving positioning performance. Simulations confirm the method’s effectiveness across various configurations.

These two recent works have shown that MIMO-OFDM signals for sensing and HR estimation shows superior sensing performance compared to Doppler and MIMO FMCW radars. While in our current work, we opted for using the latter two, the former one is a candidate for future work of ours.

## 6. Conclusions

In this paper, we proposed a heart rate estimation considering reconstructed signal features based on VMD via MIMO FMCW Radar. In the proposed method, only the signal corresponding to the human position can be used for heart rate detection by using the MIMO FMCW radar, thus reducing the effect of noise. After acquiring the signals corresponding to the human position, VMD is used to decompose the signals into multiple IMF signals, and only the IMF signals caused by heartbeat are extracted based on the center frequency of each signal. This allows the influence of components other than the heartbeat to be reduced. The reconstructed signals are then generated by weighted addition based on the energy of the multiple IMF signals, and each signal is used to estimate the heart rate based on peak detection. Finally, by selecting an appropriate estimate from the estimated heart rates in multiple cells, taking into account the time transition, the possibility of extraneous variation in the estimates may be reduced.

Experimental results showed that the proposed method achieved an average MAE of 2.54 BPM when one subject was placed within the radar coverage area, which is an improvement over the conventional method [8,9,10]. The exclusion rate of the proposed method with the Doppler radar was 4.72%, while the exclusion rate with the MIMO FMCW radar was 0.94% and detected heartbeats in a longer time.

On the other hand, an average MAE of 2.28 BPM was achieved when two subjects were placed within the radar coverage area.

As for future works, our first priority is to improve the IMF signal selection method. In this paper, IMF signals whose center frequency is within the normal heartbeat frequency range are selected. In this case, some of the reconstructed signals generated by multiple cells contain extra peaks or signals with no clear peaks. This is considered to be due to the selection of IMF signals that are not caused by heartbeats. Therefore, we believe it is necessary to select more reliable IMF signals. As a proposal to improve the IMF signal selection method, it is assumed that the IMF signal with strong periodicity is selected based on the autocorrelation function. In addition, when multiple subjects are present within the radar coverage area, signals reflected from different persons are mixed together. Since the proposed method selects IMF signals in the general heartbeat frequency range of 0.8 to 2.0 Hz, the heartbeat components of different subjects will be included in the IMF signal when the VMD extracts the IMF signal caused by heartbeats, which may degrade the accuracy of heart rate estimation. In other words, the proposed method cannot classify the heartbeat signals of multiple subjects.

In addition, when classifying the received signals of the radar to which subject each corresponds, threshold values are set for the distance and angle between the subjects to determine them. In this case, if the distance between the subjects is extremely close, the received signal cannot be classified. Furthermore, the proposed method assumes that the number of subjects is known. However, it is desirable to estimate the heartbeat without the number of subjects being known. From the above, it is necessary in the future to classify the received signals as reflected signals from each subject based on the correlation value for each signal, and then estimate the heart rate.

## Figures and Tables

**Table 1:** An example of comparison of estimation error [BPM] and reconstructed signal energy.

	Estimation Error	Reconstructed Signal Energy
cell 1	0.46	0.98
cell 2	2.83	0.74
cell 3	22.12	0.58
cell 4	2.57	0.68
cell 5	4.68	0.82
cell 6	17.74	0.70
cell 7	4.53	0.81
cell 8	4.26	0.42
cell 9	4.05	0.45
cell 10	7.83	0.61

**Table 2:** The specification of Doppler radar.

Item	Value
Modulation method	Unmodulated continuous wave
Carrier frequency	24 GHz
Sampling frequency	1000 Hz
Transmission power	1 mW

**Table 3:** The specification of MIMO FMCW radar.

Item	Value
Modulation method	FMCW
Carrier frequency	79 GHz
Bandwidth	3.43 GHz
Sampling frequency	240 Hz
Number of transmitting antennas	3
Number of receiving antennas	4

**Table 4:** The experimental specification for the heart rate estimation.

Item	Value
Distance between the radar and a subject	(i) 1.0 m, 2.0 m (ii) 2.5 m
Distance between the subjects	(ii) 0.5 m, 1.0 m
The number of subjects	(i) 4 (ii) 2
Observation duration	(i) 2 min (ii) 1 min
The threshold $th_{r}$ of the second peak to the largest peak ratio in the autocorrelation	0.6
the largest peak in the autocorrelation $th_{r}$	
Beamforming range of MIMO FMCW radar	From $-40^{\circ }$ to $40^{\circ }$
Beamforming interval of MIMO FMCW radar	$3^{\circ }$
The number of selected cells by	(i) 10
The CL method *N*	(ii) 10×numberofsubjects

**Table 5:** MAE [BPM] of estimated heart rate using MIMO FMCW radar.

	Proposed Method		Conventional Methods					
			**Spectrogram [11]**		**Viterbi [12]**		**VMD [14]**	
	**1 m**	**2 m**	**1 m**	**2 m**	**1 m**	**2 m**	**1 m**	**2 m**
subject 1	1.87	1.96	5.94	4.25	6.14	7.37	4.12	3.06
subject 2	3.96	1.31	3.99	7.17	7.59	6.78	2.23	2.96
subject 3	3.33	1.73	4.72	4.85	9.57	8.58	2.83	1.89
subject 4	2.59	4.49	3.25	4.10	7.17	8.59	5.83	4.64
Ave.	2.94	2.37	4.48	5.09	7.62	7.83	3.75	3.14

**Table 6:** MAE [BPM] of estimated heart rate using Doppler radar.

	Proposed Method		Conventional Methods					
			**Spectrogram [11]**		**Viterbi [12]**		**VMD [14]**	
	**1 m**	**2 m**	**1 m**	**2 m**	**1 m**	**2 m**	**1 m**	**2 m**
subject 1	10.17	11.86	8.99	9.09	9.85	12.51	11.23	12.07
subject 2	7.04	13.10	8.10	7.95	9.80	12.28	7.27	12.94
subject 3	6.34	13.55	9.12	9.87	7.51	12.09	6.52	13.55
subject 4	12.63	14.35	12.22	8.54	8.34	11.21	12.64	14.60
Ave.	9.04	13.22	9.61	8.86	8.88	12.02	9.42	13.29

**Table 7:** Average of true heart rate variability [BPM] in the neighboring time window.

	1 m	2 m	Ave.
subject 1	0.42	0.69	0.55
subject 2	0.87	0.52	0.70
subject 3	1.12	1.38	1.25
subject 4	0.40	0.67	0.53
Ave.	0.70	0.82	0.76

**Table 8:** Exclusion rates [%] using MIMO FMCW radar and Doppler radar.

	MIMO FMCW		Doppler	
	**1 m**	**2 m**	**1 m**	**2 m**
subject 1	0.00	3.77	13.21	5.66
subject 2	7.55	0.00	3.77	7.55
subject 3	0.00	1.89	1.89	0.00
subject 4	3.77	0.00	3.77	1.89
Ave.	2.83	1.42	5.66	3.77

**Table 9:** MAE [BPM] when the reconstructed signal is selected based on only energy or based on energy and periodicity in case (i).

	Only Energy		Energy + Periodicity	
	**1 m**	**2 m**	**1 m**	**2 m**
subject 1	4.34	2.12	1.87	1.96
subject 2	1.01	1.25	3.96	1.31
subject 3	3.26	1.79	3.33	1.73
subject 4	2.62	3.91	2.59	4.49
Ave.	2.81	2.27	2.94	2.37

**Table 10:** MAE [BPM] of estimated heart rate using MIMO FMCW radar in case (ii).

	Proposed Method		Conventional Methods					
			**Spectrogram [11]**		**Viterbi [12]**		**VMD [14]**	
	**0.5 m**	**1.0 m**	**0.5 m**	**1.0 m**	**0.5 m**	**1.0 m**	**0.5 m**	**1.0 m**
subject 1	1.84	1.51	4.68	3.55	7.63	9.53	6.33	2.63
subject 2	1.37	2.86	6.91	3.46	13.50	5.63	2.67	3.20
Ave.	1.61	2.19	5.80	3.51	10.57	7.58	4.50	2.92

**Table 11:** MAE [BPM] when the reconstructed signal is selected based on only energy or based on energy and periodicity in case (ii).

	Only Energy		Energy + Periodicity	
	**1 m**	**2 m**	**1 m**	**2 m**
subject 1	5.95	1.92	1.84	1.51
subject 2	2.33	2.64	1.37	2.86
Ave.	4.14	2.28	1.61	2.19

## Data Availability

The data presented in this study are available on request from the corresponding author due to privacy issues.

## Abbreviations

The following abbreviations are used in this manuscript:

AHC	Adaptive Harmonics Cancellation
BPF	Band-Pass Filter
BPM	Beats Per Minute
CFAR	Constant False Alarm Rate
CL	Curve-Length
CPI	Coherent Processing Interval
CW	Continuous Wave
DoA	Direction of Arrival
Double-CZT	Double-Chirp Z-Transform
ECG	Electrocardiogram
FMCW	Frequency Modulated Continuous Wave
HR	Heart Rate
ICA	Independent Component Analysis
IMF	Intrinsic Mode Function
LiDAR	Light Detection and Ranging
LPC	Linear Predictive Coding
MAE	Mean Absolute Error
MIMO	Multiple-Input Multiple-Output
RBM	Random Body Motion
RLS	Recursive least squares
RRI	R-peak to R interval
SISO	Single-Input Single-Output
SSA	Singular Spectrum Analysis
VMD	Variational Mode Decomposition
VME	Variable Mode Extraction
